# A Highly Sensitive Non-Radioactive Activity Assay for AMP-Activated Protein Kinase (AMPK)

**DOI:** 10.3390/mps1010003

**Published:** 2017-10-13

**Authors:** Yan Yan, Xin Gu, H. Eric Xu, Karsten Melcher

**Affiliations:** 1 Laboratory of Structural Sciences and Laboratory of Structural Biology and Biochemistry, Center of Cancer and Cell Biology, Van Andel Research Institute, 333 Bostwick Avenue Northeast, Grand Rapids, MI 49503, USA; eric.xu@vai.org; 2 VARI-SIMM Center, Center for Structure and Function of Drug Targets, The CAS Key Laboratory of Receptor Research, Shanghai Institute of Materia Medica, Chinese Academy of Sciences (CAS), Shanghai 201203, China; 3 University of Chinese Academy of Sciences, No. 19A Yuquan Road, Beijing 100049, China

**Keywords:** AMP-activated protein kinase (AMPK), Forkhead-associated (FHA) domain, kinase assay, AlphaScreen

## Abstract

While many methods exist to quantitatively determine protein kinase activities, ^32^P-based radioactive assays remain the workhorse of many laboratories due to their high sensitivity, high signal to noise ratio, lack of interference by fluorescent and light-absorbing small molecules, and easy quantitation. Here, we demonstrate that the interaction between the yeast Rad53 Forkhead-associated (FHA) domain and a peptide optimized for phosphorylation by AMP-Activated Protein Kinase (AMPK), which has previously been exploited for the generation of intracellular phosphorylation sensors, can serve as a readout for a highly sensitive two-step AMPK AlphaScreen kinase assay with exceptional signal-to-noise ratio.

## 1. Introduction

AMP-Activated Protein Kinase (AMPK) is a three-subunit protein kinase that functions as central cellular energy sensor and regulator of energy homeostasis in eukaryotes [1,2,3,4]. AMPK detects cellular energy states as ratios of AMP, ADP, and ATP (adenylate energy charge [5] ([ATP]+0.5x[ADP])/([ATP]+[ADP]+[AMP])) [6] by competitive binding of all three adenine nucleotides to three separate sites in its γ-subunit [6,7,8]. Energy stress, i.e., high ratios of AMP, and ADP, to ATP, strongly activate the AMPK kinase activity by multiple mechanisms [9,10,11,12,13], resulting in the phosphorylation of numerous metabolic and regulatory proteins in the cell. The net effect of these phosphorylation events is a cellular reprogramming to activate ATP-generating metabolic pathways, such as glucose and fatty acid mobilization, uptake, and catabolism, and the inhibition of ATP-consuming programs, such as the synthesis of macromolecular building blocks, growth, and proliferation [1,2,4].

AMPK activation is associated with many health benefits, making AMPK a promising target for the treatment of metabolic disorders, including diabetes, obesity, and cancer [3,14,15]. Consequently, development of therapeutic AMPK activity modulators is pursued by many pharmaceutical companies. Direct AMPK activation is determined by various kinase assays, including relatively low throughput radioactive [16,17] and HPLC-based [18] kinase assays, as well as high throughput fluorescence-based assays [4,7,19], which have relatively small signal-to-noise ratios and suffer from fluorescence interference by a substantial fraction of screening compounds. Here, we present a sensitive two-step kinase assay that is amenable to high throughput screening, has an exceptional (ca. 1000-fold) signal-to-noise ratio, and is resistant to most compound interference. This assay is based on the highly specific, phosphorylation-dependent interaction between a Forkhead-associated (FHA) domain, and an AMPK substrate peptide in an AlphaScreen assay. FHA domains are ~75 amino acid stable domains that bind Ser/Thr-phosphorylated peptides [20,21,22], a binding reaction that has recently been exploited for the generation of intracellular phosphorylation sensors for several protein kinases, including AKT [23], Protein kinase A (PKA) [24], ataxia-telangiectasia mutated (ATM) kinase [25], and AMPK [26]. By separating AMPK substrate phosphorylation reactions in the absence or presence of AMPK activity modulators from a subsequent AlphaScreen phospho-substrate/FHA interaction assay, the assay becomes highly robust against compound interference and allows for the determination of absolute enzyme activity at substrate concentration at or above Michaelis constant (Km). Given the prominent roles of protein phosphorylation in signal transduction and of protein kinases as drug targets, this assay can also be adapted to determine the activities of other kinases.

## 2. Materials and Methods

### 2.1. Protein Expression and Purification

All of the expression constructs were confirmed by DNA sequencing. All of the proteins were expressed from the tricistronic *Escherichia coli* expression vectors described previously [27,28] H6-α_1_(13-550)-β_1_(68-270; S108D)-γ_1_-AMPK, maltose-binding protein (MBP)-α_1_(13-550)-β_1_(68-270; S108D)-γ_1_(24-327)-AMPK, and MBP-α_1_(11-550)-β_2_-γ_1_-AMPK expression plasmids were transformed into *E. coli* BL21 (DE3) (AMPKα1(13-550): NCBI/GenBank: AAH37303.1; AMPKβ1(68-270): UniProt ID Q9Y478, NCBI: NP_006244.2; AMPKβ2: UniProt ID O43741; AMPKγ1: NCBI: NP_002724.1. His6-AMPK and MBP-AMPK exhibit identical fold activation by AMP and pharmacological activators, but MBP-AMPK has slightly higher total kinase activity. Cells were grown in LB medium (1% w/v tryptone, 0.5% w/v yeast extract, 1% w/v NaCl) to an OD_600_ of ~1 at 28 °C and induced with 100 μM isopropyl-β-D-thio-galactopyranoside (IPTG) at 16 °C overnight. Cell pellets were resuspended in His/MBP buffer A (His buffer A: 25 mM Tris, pH 8.0, 300 mM NaCl, 25 mM imidazole, 10% glycerol, 5 mM β-mercaptoethanol; MBP buffer A: 25 mM Tris, pH 8.0, 300 mM NaCl, 5 mM MgCl_2_, 1 mM ethylenediaminetetraacetic_acid (EDTA), 10% glycerol, 2 mM dithiothreitol (DTT), and lysed by French Press with pressure set to 900 Pa. Lysates were cleared by centrifugation for 30 min at 20,000× *g* at 4 °C, passed over a 10 mL HisTrap HP column/MBPTrap HP column (GE Healthcare), and eluted with His/MBP buffer B (His buffer B: 25 mM Tris, pH 8.0, 300 mM NaCl, 500 mM imidazole, 10% glycerol, 5 mM β-mercaptoethanol; MBP buffer B: 25 mM Tris, pH 8.0, 300 mM NaCl, 40 mM maltose, 5 mM MgCl_2_, 1 mM EDTA, 10% glycerol, 2 mM DTT). The eluted AMPK was further purified by size-exclusion chromatography through a HiLoad 26/60 Superdex 200 column (GE Healthcare) in 25 mM Tris, pH 8.0, 300 mM NaCl, 5 mM MgCl_2_, 1 mM EDTA, 10% glycerol, 2 mM DTT. Phosphorylated AMPK was generated by incubation with 0.02-fold molar ratio of CaMKKβ in 0.2 mM AMP, 0.2 mM ATP, 2 mM CaCl_2_, 10 mM DTT, and 1 μM calmodulin at room temperature overnight (16 h). The phosphorylated AMPK was further purified by size-exclusion chromatography through a HiLoad 26/60 Superdex 200 column (GE Healthcare) in 25 mM Tris, pH 8.0, 300 mM NaCl, 5 mM MgCl_2_, 1 mM EDTA, 10% glycerol, 2 mM DTT. The His6GST-FHA purification protocol is the same as for His-tagged AMPK. The FHA domain corresponds to *Saccharomyces cerevisiae* Rad53(22-162) (NCBI Reference Sequence: NP_015172.1). The protein eluted from the gel filtration column at a volume corresponding to the size of a monomeric complex at a purity ≥95% as judged by sodium dodecyl sulfate polyacrylamide gel electrophoresis (SDS-PAGE) (Figure S1).

### 2.2. AMPK AlphaScreen Kinase Assay

10 nM AMPK were incubated with 50 μM b-ASP (biotinylated AMPK substrate peptide: biotin-GSTKMRRVA**T**LVDLGYKK; synthesized by Peptide 2.0 Inc., Chantilly, VA, USA) and 100 µM ATP in kinase buffer (25 mM Tris, pH 8.0, 300 mM NaCl, 5 mM MgCl_2_, 1 mM EDTA, 10% glycerol, 2 mM DTT) in a microcentrifuge tube in a total volume of 10 μL for 20 min at room temperature (non-phosphorylated AMPK) or on ice (phosphorylated AMPK). The reaction was terminated by a two-step 1000-fold dilution by adding 490 μL kinase buffer (50-fold dilution), of which 7.5 μL were added to a total volume of 150 μL in AlphaScreen buffer (50 mM MOPS, pH7.4, 50 mM NaF, 50 mM CHAPS, and 0.1 mg/mL bovine serum albumin) containing 50 nM His6GST-FHA, 5 μg/mL AlphaScreen Streptavidin-coated Donor beads and 5 μg/mL Nickel-chelate Acceptor beads (PerkinElmer, Waltham, MA, USA). AlphaScreen reactions were incubated for 1.5 h in the dark at room temperature. Donor and acceptor beads are brought into proximity by the interaction between b-^p^ASP and His6GST-FHA. When excited by a laser beam of 680 nm, the photosensitizer in the donor beads converts ambient oxygen into short-lived singlet oxygen that can transfer energy to the thioxene derivatives in the acceptor beads, releasing photons of 520–620 nm as the binding signal that can be measured in an Envision (PerkinElmer) plate reader.

For absolute activity determinations, nM p-ASP in the AlphaScreen reaction was determined by interpolation from the calibration curve using the “Interpolate unknowns function by non-linear regression” of GraphPad Prism. Since the AlphaScreen reaction was performed with 1/1000th of the , total first step kinase reaction (1000-fold dilution), nM p-ASP in the AlphaScreen reaction corresponds to µM substrate conversion in the first step kinase reaction. The kinase reaction contains 0.01 μM AMPK and proceed for 20 min, absolute activities are therefore 1 μM p-ASP per 0.01 μM AMPK per 20 min for every 1 nM p-ASP in the AlphaScreen reaction, i.e., each nM p-ASP in the AlphaScreen reaction corresponds to 100 µmol substrate conversion/(μM AMPK × 20 min) = 5 μmol substrate conversion × (µmole AMPK)^−1^ × min^−1^ (which corresponds to 0.6 nmol mg^−1^ AMPK min^−1^ for H6-AMPK (120 kDa)).

### 2.3. AMPK [^32^P]-γATP Kinase Assay

For the radioactive kinase assay, 10 nM AMPK were incubated with 50 μM b-ASP, 2 mM DTT, and 0.25 μL [^32^P]-γATP per 15 µL reaction in kinase buffer (25 mM Tris, pH 7.4, 12 mM MgCl_2_, 1 mM Na_3_VO_4_, 5 mM NaF) for 30 min at room temperature. Reactions were terminated by addition of 0.5 volumes of 7.5 M guanidine hydrochloride solution in water and reactions were spotted on a SAM 2^®^ Biotin Capture Membrane (Promega, Madison, WI, USA). The membrane was washed once for 30 s with 2 M NaCl, 3 times for 2 min with 2 M NaCl, 4 times for 2 min with 2 M NaCl in 1% H_3_PO_4_, and two times for 30 s with deionized water to remove unbound reaction components. Membranes were dried at room temperature for 30–60 min, and signals were quantitated by PhosphorImager analysis.

## 3. Results and Discussion

### 3.1. The FHA Domain Can Highly Selectively Distinguish Phosphorylated from Non-Phosphorylated AMPK Substrate Peptide

**Figure 1:**
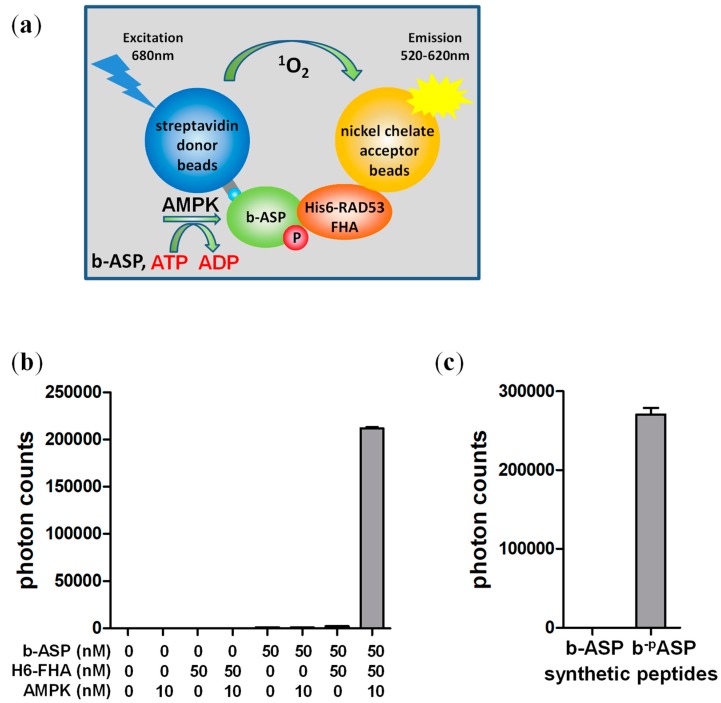
**One-step AlphaScreen kinase assay.** (**a**) Schematic presentation of the assay. Incubation of streptavidin donor bead-bound biotinylated version of the AMPK substrate peptide (b-ASP) with H6-α1(13-550)-β1(68-270; S108D)-γ1-AMPK leads to b-ASP phosphorylation, which in turns allows b-ASP to bind H6-FHA immobilized to Ni-charged acceptor beads. This interaction brings acceptor and donor beads into close proximity to generate an amplified, singlet oxygen-mediated luminescence proximity signal; (**b**) AMP-Activated Protein Kinase (AMPK) mediates a robust AlphaScreen luminescence signal that is dependent on b-ASP and His6-FHA; (**c**) Non-phosphorylated b-ASP does not interact with the Forkhead-associated (FHA) domain in the AlphaScreen assay. Number of replicates = 3, error bars = standard deviation. ADP: adenosine diphosphate, ATP: adenosine triphosphate.

AMPKAR is a FRET-based AMPK activity sensor that detects the phosphorylation-dependent intramolecular interaction between an enhanced yellow fluorescent protein (eCFP)-tagged yeast Rad53 FHA domain and an optimized Venus-tagged AMPK substrate peptide in the context of an eCFP-FHA-peptide-Venus fusion protein [26]. To test whether the FHA/phospho-peptide interaction can be utilized in trans (inter-molecularly) for quantitative determination of AMPK kinase activity, we synthesized a biotinylated version of the AMPK substrate peptide (b-ASP; Figure 1a) and expressed and purified the FHA domain as His6GST-fusion protein. We then subjected b-ASP and His6GST-FHA in a standard kinase buffer in the presence and absence of non-phosphorylated α_1_β_1_γ_1_-AMPK to a one-step combined kinase and AlphaScreen reaction. In this reaction, AMPK phosphorylates streptavidin donor bead-bound b-ASP to b-^p^ASP, which interacts with Ni acceptor bead-bound His6GST-FHA to bring donor and acceptor beads into close proximity for generation of an amplified, singlet oxygen mediated luminescence signal (Figure 1a). Even though non-phosphorylated AMPK has less than 1% of the activity of phosphorylated AMPK [29,30], we observed a strong luminescence signal that was dependent on both interaction partners, b-ASP and His6GST-FHA, as well as on AMPK (Figure 1b), suggesting that the b-ASP/FHA interaction requires phosphorylation of the central threonine by AMPK. We further validated the phosphorylation requirement using a synthetic phospho-threonine containing b-^p^ASP peptide (biotin-GSTKMRRVA^p^**T**LVDLGYKK) and tested the luminescence interaction signal of both phosphorylated and non-phosphorylated peptide side by side. As shown in Figure 1c, b-^p^ASP interacted strongly with the FHA domain, whereas no interaction was detectable with the non-phosphorylated b-ASP peptide.

### 3.2. A Two-Step Assay Allows Determination of Absolute AMPK Kinase Activities

**Figure 2:**
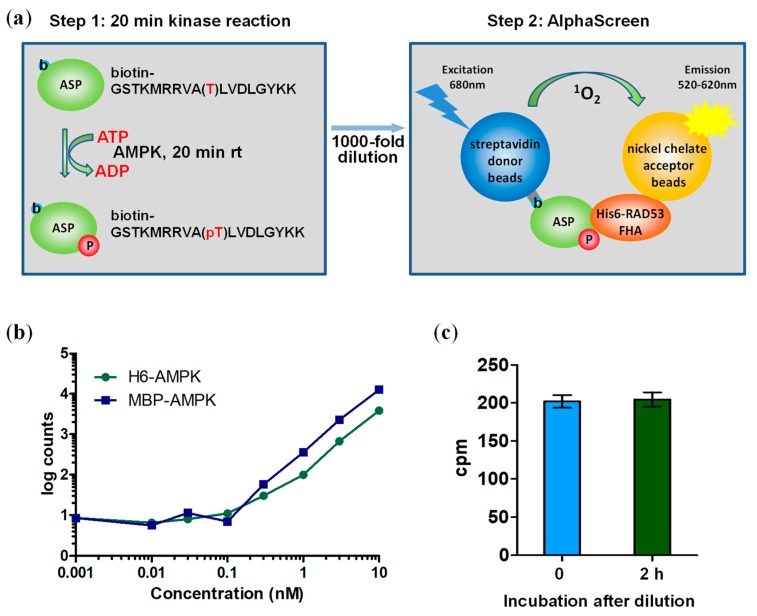
**Two-step AlphaScreen kinase assay.** (**a**) Schematic assay presentation. (**b**) AMPK is inactive when diluted to ≤0.1 nM. Activities were determined by standard radioactive peptide assay. (**c**) 1000-fold dilution terminates the kinase (Step 1) reaction. The Step 1 reaction was performed in the presence of ^32^P-γATP, diluted 1000-fold, and then either directly spotted on streptavidin-coated filter paper or after a two hour additional incubation in the diluted kinase buffer. ^32^P incorporation into the b-ASP peptide was determined by PhosphorImager analysis. Number of replicates = 3, error bars = standard deviation; rt: room temperature.

The streptavidin donor beads have a b-ASP binding capacity of about 50 nM, a concentration that is two orders of magnitude below the Km of the reaction and leads to substrate exhaustion. We therefore set up a two-step assay (Figure 2a) to allow in the first step the kinase reaction to proceed at substrate concentrations (ATP: 100 μM; b-ASP peptide: 50 μM) above Km (Km ATP: 26–35 μM; Km of less optimal AMPK substrate peptide SAMS: 27 μM) [4,31], and then dilute the reaction 1000-fold to achieve a low concentration (50 nM) of total b-ASP (b-ASP + b-^p^ASP) that matches the streptavidin donor bead capacity during the subsequent (second step) Alphascreen reaction in 384 well plates. Because AMPK phosphorylates b-ASP more efficiently than the commonly used SAMS peptide substrate, it was important to establish conditions at which AMPK does not cause substrate exhaustion. While the simplest way to limit b-ASP phosphorylation is by AMPK dilution, we found that the AMPK heterotrimer becomes unstable and loses catalytic activity at concentrations ≤100 pM (Figure 2b). We therefore kept the concentration of AMPK constant at 10 nM and incubated for 20 min either at room temperature (non-phosphorylated AMPK) or on ice (phosphorylated AMPK), conditions at which AMPK does not cause substrate exhaustion and remains completely stable. 1000-fold dilution efficiently and immediately terminates the reaction (Figure 2c) due to the instability of AMPK at 10 pM concentration and due to concentrations of ATP and b-ASP that are about two orders of magnitude below Km.

**Figure 3:**
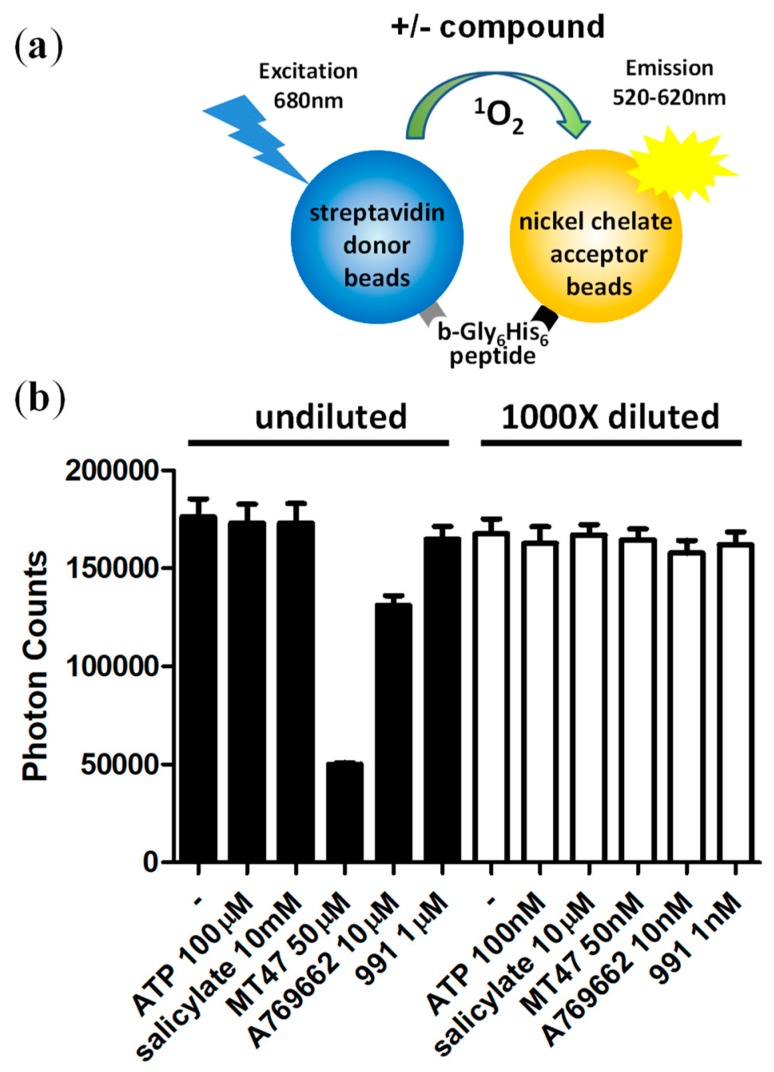
Reaction dilution abolishes signal interference by pharmacological AMPK activators. (**a**) Schematic of the control AlphaScreen reaction; (**b**) MT47-100 and A769662 interfere with the luminescence signal at concentrations used in kinase assays. No interference is detectable at 1000-fold diluted compound concentrations. Number of replicates = 3, error bars = standard deviation.

An additional advantage of the two-step protocol is that it allows the performing of the reaction in the presence of most fluorescent and/or light-absorbing compounds, which represent a significant fraction of molecules in common drug screening compound libraries. To test whether AMPK-modulating compounds interfere with the AlphaScreen signal, we incubated a biotinylated Gly_6_His_6_ control peptide (b-Gly_6_His_6_; binds simultaneously acceptor and donor beads) with ATP and prominent pharmacological AMPK activators at concentrations at which they are typically used in AMPK kinase assays as well as at 1000-fold dilution of the kinase reactions. While MT47-100 [32] and A769662 [33] clearly interfered with the assay at 50 μM and 10 μM, each, none of the AMPK modulators interfered with the signals in the 1000-fold diluted reactions, as was expected (Figure 3).

### 3.3. Assay Normalization

**Figure 4:**
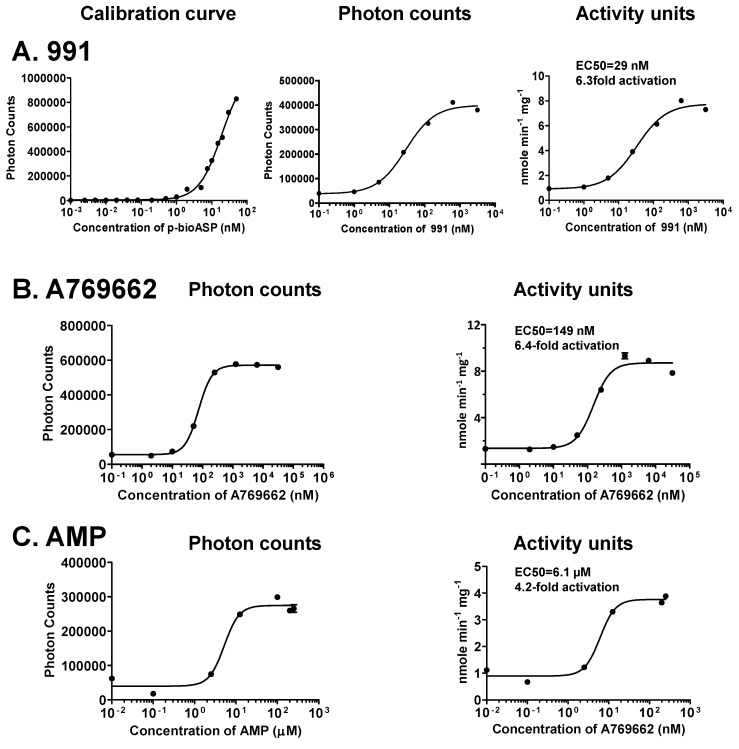
Determination of absolute kinase activities of non-phosphorylated H6-α1(13-550)-β1(68-270; S108D)-γ1-AMPK calibration. (**a**) 991; (**b**) A769662; (**c**) AMP. *N* = 3, error bars = SD.

To calibrate the assay for quantitation, we generated Alphascreen dose-response curves using 50 nM b-ASP at defined ratios of non-phosphorylated and phosphorylated peptide. As seen in Figure 4, we detected steep dose-response curves, allowing for accurate activity measurements within the b-^p^ASP concentration range between initial signal increases and the beginning signal saturation. Since this range is tight, it is important to include a calibration data set on the same plate that contains the test data. In those cases where test data fall outside of the variable range of the calibration curve, assay conditions need to be adapted by changes in incubation time or dilution. As a test case we generated dose-response curves for the pharmacological activators 991 [34] (Figure 4A) and A769662 [33] (Figure 4B), as well as for the physiological activator AMP (Figure 4C). Given the different protein preparations, EC50 values and fold activation are in similar range as published for radioactive assays using the SAMS peptide as substrate (991: EC50 = ~80 nM, 11-fold activation [34]; A769662: EC50 = ~80 nM, 2.7-fold activation; AMP: EC50 = ~3 μM, 3.7-fold activation for non-phosphorylated AMPK [30,35].

### 3.4. Sensitivity of the Forkhead-associated-AlphaScreen Assay Is Comparable to that of a Radioactive Kinase Assay

**Figure 5:**
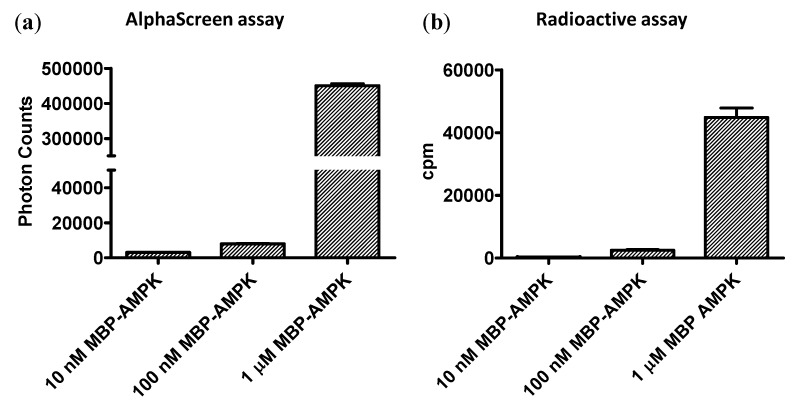
Side-by-side sensitivity comparison of AlphaScreen and radioactive kinase assays. Indicated concentrations of non-phosphorylated MBP-α_1_(11-550)-β_2_-γ_1_-AMPK were incubated with b-ASP for 10 minutes on ice and phosphoryl transfer determined by Two-step AlphaScreen kinase assay (**a**) or by PhosphorImager quantitation of the radiolabel transfer (**b**). *N* = 3, error bars = SD.

Currently, radioactive kinase assays are the gold standard for assay sensitivity. To compare the sensitivities of the AMPK FHA-AlphaScreen assay with a [^32^P]-ATP kinase assay, we incubated 10 nM, 100 nM, and 1 μg low-activity non-phosphorylated MBP-α1β1γ1-AMPK in the presence or absence of ^32^P-ATP with b-ASP substrate for 10 min on ice (to minimize kinase activity). We captured b-ASP from the radioactive kinase assay on streptavidin-coated filters and exposed them to a PhosphorImager screen either for 4 h or overnight. b-ASP from the non-radioactive reaction was 1000-fold diluted and analyzed in the AlphaScreen assay. As shown in Figure 5, both the AlphaScreen assay and the radioactive assay had similar sensitivity limits and both were clearly capable to detect substrate phosphorylation in a reaction with 100 ng non-phosphorylated AMPK at 4 °C, conditions under which AMPK has very low activity. While, the total PhosphorImager counts were higher after overnight exposure, fold signal changes were actually larger after 4 h exposure (shown in Figure 5b).

## 4. Conclusions

In this paper, we present a highly sensitive non-radioactive AMPK kinase assay that can be performed for high-throughput screening in 384-well plates. This assay is very affordable as it does not depend on commercial antibodies and/or expensive fluorescent tracers, but rather uses recombinant FHA protein that can be expressed and purified from *E. coli* in large quantities with minimal effort. A hallmark of this assay is its exceptional signal-to-noise ratio due to the steep response curve, which makes it ideal for highly sensitive compound screening. However, the steep response limits linearity of the assay to a relatively small product concentration range, which makes parallel determination of a calibration curve essential. Quantitative determination requires that all of points of the dose-response curve fall within the linear range of the calibration curve. Since this can be challenging, together with the high sensitivity of the assay, it is best suited for initial high throughput compound screening. We note that while this assay has been specifically designed for determination of AMPK activity, it can in principle be adapted to any protein kinase.

## Supplementary Materials

The following are available online at  www.mdpi.com/2409-9279/1/1/3/s1, Figure S1: Size exclusion chromatography and SDS PAGE profiles of purified proteins.
