# Supplementary material for: A Highly Sensitive Non-Radioactive Activity Assay for AMP-Activated Protein Kinase (AMPK)
Source: Methods Protoc. 2017 Oct 13;1(1):3. doi: 10.3390/mps1010003 (PMC5809138; doi:10.3390/mps1010003)
Supplement: Supplementary file 1 [file mps-01-00003-s001.pdf]

**Yan Yan[1,2,*], Xin Gu[1,*], H. Eric Xu[1,2], and Karsten Melcher[1].**

# A highly sensitive and robust non-radioactive activity assay for AMP-activated protein kinase (AMPK)

## Supplementary Figures

**Supplementary Figure 1. Size exclusion chromatography and SDS PAGE profiles of purified proteins. (A)** H6GST-FHA[Rad53(22-162)], **(B)** H6-$\alpha_1$(13-550)-$\beta_1$(68-270; S108D)-$\gamma_1$-AMPK, and **(C)** MBP-$\alpha_1$(13-550)-$\beta_1$(68-270; S108D)-$\gamma_1$(24-327)-AMPK. Black line: non-phosphorylated (non-P) AMPK; red line: phosphorylated AMPK (P).

# Fig. S1

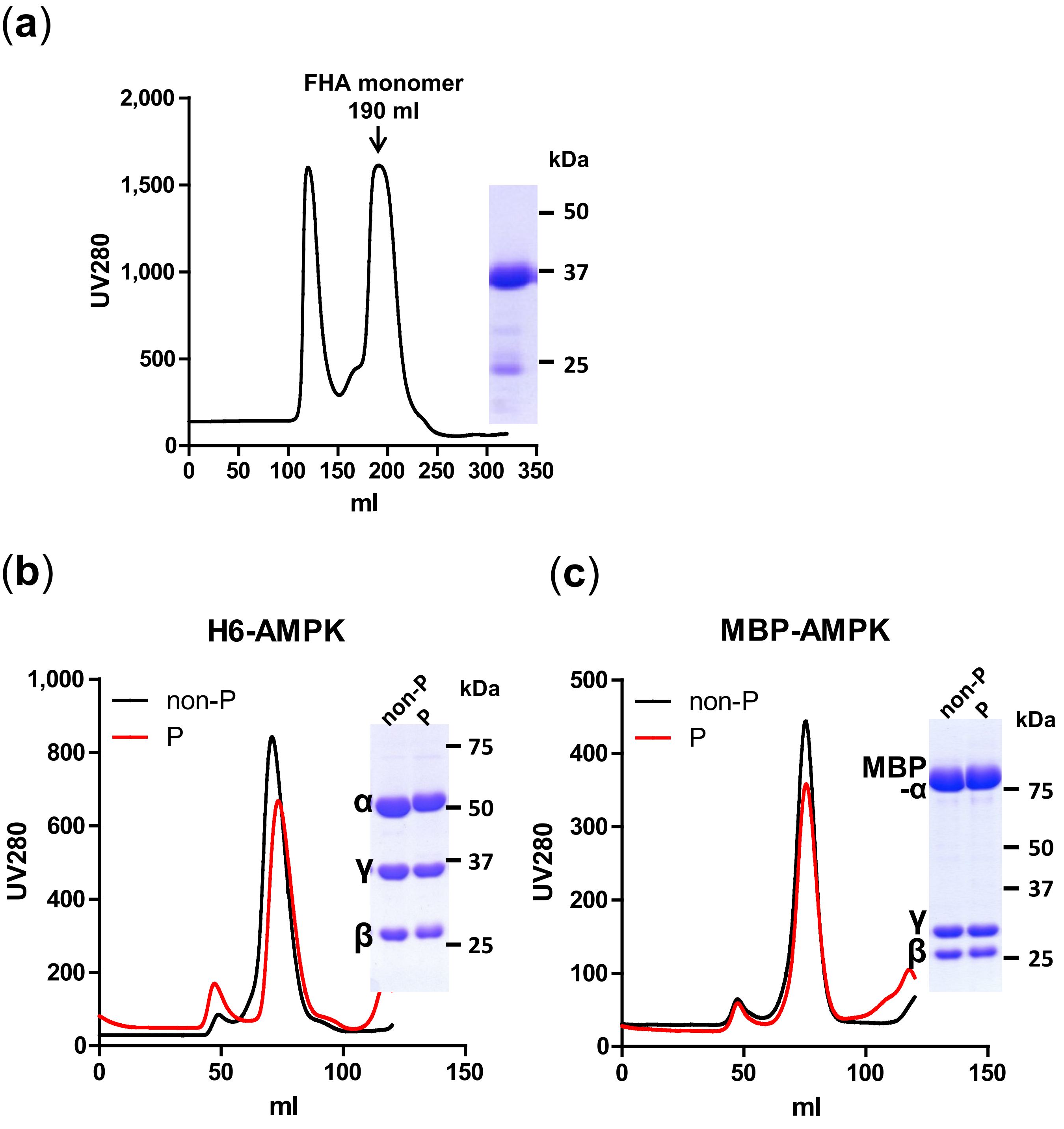
(a)
FHA monomer
190 ml
kDa
50
37
25
UV280
2,000
1,500
1,000
500
0
0
50
100
150
200
250
300
350
ml
(b)
H6-AMPK
non-P
P
non-P
P
kDa
75
50
37
25
α
γ
β
UV280
1,000
800
600
400
200
0
0
50
100
150
ml
(c)
MBP-AMPK
non-P
P
non-P
P
kDa
MBP
-α
75
50
37
25
γ
β
UV280
500
400
300
200
100
0
0
50
100
150
ml
